# Medicine prices, availability and affordability in Sri Lanka

**DOI:** 10.4103/0253-7613.75672

**Published:** 2011-02

**Authors:** S.M.D.K. Ganga Senarathna, Uthpali Mannapperuma, B.M. Rohini Fernandopulle

**Affiliations:** Department of Pharmacology, Faculty of Medical Sciences, University of Sri Jayawardenapura, Nugegoda, Sri Lanka; 1 Department of Pharmacology, Faculty of Medicine, University of Colombo, Colombo, Sri Lanka

**Keywords:** Affordability, availability, medicine prices, price control

## Abstract

**Background:**

No pricing formula has been implemented from November 2002 to date in Sri Lanka. Therefore, we initiated a study in 2003 to determine the prices, availability and affordability of medicines in the private sector of Sri Lanka in the absence of a price control.

**Materials and Methods:**

The World Health Organization/Health Action International methodology was used. The study was conducted in retail pharmacies (*Rajya Osu Sala*) of State Pharmaceuticals Corporation (semigovernment) and privately owned retail pharmacies (*n* = 15) in 2003, 2006 and 2009 in a geographical area. Essential medicines (*n* = 28) were studied and, for each medicine, innovator, most sold generic and cheapest generic were monitored. The medicine’s median price was compared with the international reference prices (IRP) to obtain the median price ratio. The daily wage of the lowest-paid government worker was used to calculate affordability.

**Results:**

Innovators were five to six-times the IRP at privately owned pharmacies and four to seven-times at the *Rajya Osu Sala*. The prices of generics were ≤1 the IRP during 6 years in privately owned and *Rajya Osu Sala* pharmacies. Cheapest generics were high in availability (>80%) throughout the study period. Innovators cost more than a day’s wage of the lowest-paid government worker; in contrast, generics were always less than one day’s wage. There seems to be no difference in affordability between privately owned or semigovernment pharmacies.

**Conclusion:**

In Sri Lanka, generic medicines have effective pricing and are available and affordable. No drastic changes in prices of medicine in the private sector were observed over the 6 years despite removal of price control.

## Introduction

Health care in Sri Lanka is delivered by both government and private sectors. Medicines are free at the point of delivery at the government sector. However, it is estimated that the private sector accounts for between 50 and 60% of out patient care.[1] In 2002, consumers have paid out-of-pocket 16.9 billion Sri Lankan rupees to purchase medicines for outpatient care in the private sector in contrast to 516 million Sri Lankan rupees by the government sector outpatient care.[2] Price is one of the significant determinants of the cost and purchasing power of medicines in the private sector.[3]

The government of Sri Lanka has imposed price control on pharmaceuticals from time to time. The last was the pricing formula by the Fair Trading Commission, where the retail price was fixed at a maximum of 160% of the cost, insurance and freight by the Sri Lanka Government Gazette Extraordinary No. 552/7 in 1989. No new pricing formula has been introduced since the announcement of its termination by the Sri Lanka Government Gazette Extraordinary No.1259/14, with effect from November 2002.

Most developed countries other than the USA practice direct and indirect pricing policies with the common objective of achieving affordability of medicines. The direct pricing policies include negotiated prices, maximum fixed prices, international price comparisons and price cuts or freezes. Indirect methods include profit regulation and reference or index pricing.[4] However, a recent Cochrane systematic review on the effects of medicine pricing policies could not find any studies from developing countries that could inform decision-making.[5]

It is important to measure the price of medicines as the first stage in developing a medicine pricing policy. Further, Sri Lanka is currently planning on a National Drug Policy, and medicine prices would be one of the areas of importance in this context.

The objectives of this study were to determine the prices of medicines, the pattern of change in prices over a 6-year period, the variation of prices between pharmacies at a time in a given area, the availability of medicines and affordability of medicines in the absence of a pricing formula in the private sector of Sri Lanka.

## Materials and Methods

The standardized methodology developed by the World Health Organization (WHO) and Health Action International (HAI) was used.[3] The study was conducted in one geographical area in 15 pharmacies.

In Sri Lanka, the retail pharmacy sector comprises of privately owned pharmacies and retail pharmacies (*Rajya Osu Sala*) of the State Pharmaceuticals Corporation (semigovernment). The procurement system of the State Pharmaceuticals Corporation is a worldwide tendering system, which is open to the world. The retail pharmacies of the State Pharmaceuticals Corporation practice a chain pharmacy concept, and prices and availability of medicine are uniform in all *Rajya Osu Sala* in the country and they are located in most parts of the country. The study was carried out in both these categories of pharmacies; *Rajya Osu Sala* and privately owned pharmacies.

A total of 28 medicines were included and the selection criteria were the local burden of disease and their inclusion in the essential medicines list of Sri Lanka.[6] The selected drugs also needed to have international reference prices (IRP).[7]

For each medicine, three products were monitored: innovator brand (INO), most sold generic equivalent (MSG) and cheapest generic equivalent (CG). The MSGs were selected using International Management Sciences for Health (IMS) data, which provides details of market sales of medicines of different countries, including Sri Lanka (Balasubramaniam M.; Personal communication). The CGs were determined on the spot.

Data were collected using the standardized medicine price data collection form. The collected data were entered into the software, international medicines price work book.[3]

For the purpose of analysis, the median price ratios (MPRs), which is the ratio between the median local unit price of the medicine to patient (in US$), and the medicine’s IRP was taken. The work book software calculates the MPRs only if the medicines are found in more than four pharmacies.[3] The MPR expresses how much greater or lesser the local medicines prices are as compared with the IRP.[3]

The IRP was obtained from the Management Sciences for Health Medicine Price Indicators for 2003, 2006 and 2009.[7] The IRP for medicines is the procurement prices for generic medicines offered to countries of low and middle income by nonprofit making suppliers and agencies. IRPs are periodically updated and published by the Management Science for Health.[7] The exchange rates were obtained from the Central Bank of Sri Lanka.[8–10] The summary MPR is the median of MPRs of all INO, MSGs and CGs.[3]

The brand premium calculates how large the INO prices are from its generics (the MSG or the CG). The brand premium was calculated by pairing up brand with its generics (INOs with its MSG and INOs with its CG) and dividing the summary MPR of INOs with the summary MPR for generics of matching pairs of medicines.[3]

To assess affordability, five common diseases in Sri Lanka were selected. The affordability was computed using the daily wage of the lowest paid unskilled government worker. The salary scales of the government sector were revised on April 25, 2006, increasing the basic salary 2.5-times by the Public Administration Circular of the Government of Sri Lanka No. 6/2006.

## Results

### 

#### Medicine prices

In privately owned pharmacies, the summary MPRs of 19 innovator brands were 4.72-times the IRP, while INOs for acyclovir, diazepam, mebendazole and omeprazole were more than 20-times the IRP in 2009. Median price ratios of innovator medicines in the three study periods are given in Table 1. The 22 most sold generics and cheapest generic products, summary MPRs were 0.75 and 0.44 in 2009.

**Table 1:** MPRs of innovator medicine in the privately owned pharmacies in 2003, 2006 and 2009

*Medicine*	*MPR*		
	*2003*	*2006*	*2009*
Aciclovir 200 mg	14.83*	-	25.51*
Amitriptyline 25 mg	4.84	3.81	4.72
Amoxicillin 250 mg	6.22	5.58	3.84
Atenolol 50 mg	20.03#	21.48#	8.53#
Carbamazepine 200 mg	9.43	7.58	7.80
Diazepam 5 mg	32.14±	16.74±	19.97±
Enalapril 5 mg	9.34	9.34	6.85
Erythromycin 250 mg	2.71	3.77	3.11
Frusemide 40 mg	3.58	3.51	3.28
Glibenclamide 5 mg	3.72	6.23	4.01
Mebendazole 100 mg	26.73Π	34.54Π	21.30Π
Metformin 500 mg	3.00	5.32	2.43
Omeprazole 20 mg	5.73Ψ	-	24.62Ψ
Ranitidine 150 mg	1.99	7.10	5.09
Salbutamol inhaler (MDI) 0.1 mg/dose	2.09	2.11	1.65

* Increase in MPR of aciclovir is mostly due to drop in IRP from 0.0854 to 0.0551;

# Drop in MPR of atenolol in 2009 is mostly due to rise in IRP in 2009 to 0.0161 from 0.0082 (2003) and 0.0088 (2006);

± Drop in MPR of diazepam from 2003 to 2009 is mostly due to rise in IRP from 0.0029 (2003) to 0.0042 (2006) and 0.005 (2009);

Π Change in MPR is mostly due to change in median price of mebendazole;

Ψ Rise in MPR of omeprazole is mostly due to drop in IRP from 0.1845 (2003) to 0.0328 (2009)

**Figure 1:**
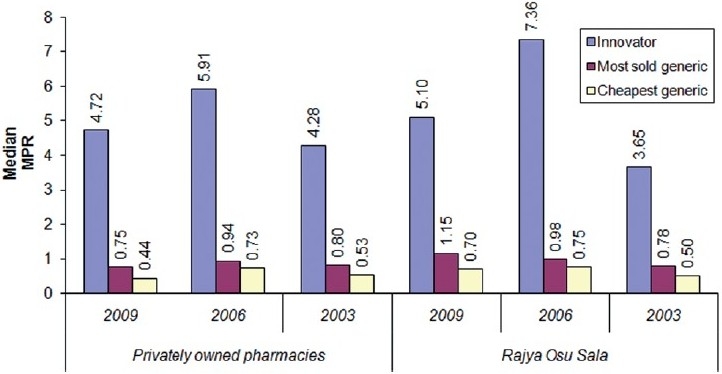
Comparison of summary median price ratios for privately owned pharmacies and *Rajya Osu Sala* in 2003, 2006 and 2009

In the State Pharmaceuticals Corporation’s retail outlet *Rajya Osu Sala*, the summary MPRs of 15 innovator brands were 5.1-times the international reference price while for 21 most sold generics and 28 cheapest generic products, the summary MPRs were 1.15-times and 0.70-times more, respectively in 2009. The summary MPRs of the privately owned retail pharmacies and *Rajya Osu Sala* during the study periods are given in Figure 1.

#### Variation of prices among pharmacies

Price variation of a medicine among privately owned pharmacies within the same area were determined by recording the maximum and the minimum MPRs of the innovator products in 2009.

The highest price variation was for mebendazole 100 mg tablet, with a maximum MPR of 23.24 and a minimum of 20.02 per tablet, followed by carbamazepine 200 mg tablet (7.90 maximum and 5.08 minimum MPR per tablet) and omeprazole 20 mg tablet (25.10 maximum and 23.26 minimum MPR per tablet). Other innovator products surveyed showed a difference of <1 MPR per unit dose.

#### Brand premium

The brand premium between INO and MSG was 6.58 in 2003 (pairs = 14, MPRs; INO = 5.53, MSG = 0.84), 5.88 in 2006 (pairs = 16, MPRs; INO = 6.23, MSG = 1.06) and 6.71 in 2009 (pairs = 14, MPRs; INO = 4.9, MSG = 0.73). The brand premium between INO and CG was 8.12 in 2003 (pairs = 18, MPRs; INO = 5.28, MSG = 0.65), 8.10 in 2006 (pairs = 16, MPRs; INO = 5.91, MSG = 0.73) and 8.45 in 2009 (pairs = 18, MPRs; INO = 4.9, MSG = 0.58) in privately owned pharmacies.

Among the 28 medicines selected, there were two exceptions in 2006, where MSG products of cephalexin (1.2-times) and glibenclamide (five-times) were higher in price than that of the INO. This trend could not be studied in 2009 as the most sold generic and the cheapest generic products of these medicines were not available in more than four pharmacies to calculate the MPR.

When brand premium of individual medicines are considered, diazepam had the highest brand premium in all three study periods. The innovator product continued to be more than 100-times the MPR of the cheapest generic product (164.55 in 2009, 108.5 in 2006 and 150.83 in 2003).

It was also noted that brand premiums were of the same ratio for certain products in 2003 and 2009. The INO/MSG was the same in 2003 and 2009 for acyclovir (5.47), amitriptyline (8.23), amoxicillin (5.97), atenolol (44.93), carbamazepine (13.00) and mebendazole (15.20). The INO/CG brand premiums were the same for amoxicillin (6.14), enalapril (22.41), mebendazole (53.20), metformin (12.38), metronidazole (8.46) and omeprazole (60.06).

#### Availability

In privately owned pharmacies, the median availability of innovator products was 50% throughout. The median availability of the cheapest generic equivalents was more than 75% in all the three study periods. The most sold generics showed a median availability of 60% in 2003 and 2009 while showing 80% median availability in 2006.

The innovator products of ciprofloxacin, cloxacillin, hydrochlorothiazide, nifedipine retard, ORS, prednisolone and verapamil were not available in the pharmacies surveyed in all three study periods. During the study periods, the cheapest generic equivalents of erythromycin and omeprazole recorded 100% availability in all private sector pharmacies surveyed.

#### Affordability

Monthly hypertensive treatment with the innovator brand atenolol (50 mg once daily) required 3.09 days wages in 2003, 1.48 days wages in 2006 and 1.22 days wages in 2009 in privately owned pharmacies. The same treatment only cost 0.07 days wages in 2003, 0.05 days in 2006 and 0.03 days in 2009 for the most sold generic product. The cheapest generic products cost 0.07 days wages in 2003, 0.04 days wages in 2006 and 0.03 days wages in 2009.

A month’s treatment of epilepsy with INO carbamazepine (200 mg thrice daily) cost 17.1 days wages in 2003 while it was 5.16 in 2006 and 6.78 days in 2009. The most sold generic equivalents and cheapest generics cost 1.32 days salary in 2003, whereas it was half days salary for 2006 and 2009 in private pharmacies.

It was noted that the affordability in terms of day’s wages of lowest paid unskilled government worker was not affected by the place of purchase. One-month treatment of INO carbamazepine (200 mg thrice daily) for epilepsy in 2009 required 5.70 days wages at the *Rajya Osu Sala* and 6.78 days wages at fully owned private pharmacies. Table 2 gives affordability of selected medicine for few common diseases at privately owned pharmacies.

**Table 2:** Affordability of selected medicines in 2003, 2006 and 2009 in privately owned pharmacies

*Medicine (duration and disease condition)*	*Year*	*Innovator*	*Most sold generic*	*Cheapest generic*
Atenolol 50 mg tablet (monthly treatment of hypertension)	2003	3.09	0.07	0.07
	2006	1.48	0.05	0.04
	2009	1.22	0.03	0.03
Amoxicillin 250 mg capsule (1-week treatment of acute respiratory infection)	2003	1.46	0.63	0.58
	2006	0.63	0.10	0.10
	2009	0.58	0.10	0.09
Metformin 500 mg tablet (monthly treatment of diabetes)	2003	3.83	1.85	0.31
	2006	1.78	0.80	0.12
	2009	1.52	0.73	0.12
Carbamazepine 200 mg tablet (monthly treatment of epilepsy)	2003	17.10	1.32	1.32
	2006	5.16	0.54	0.54
	2009	6.78	0.52	0.52

## Discussion

### 

#### Medicine prices

Innovator medicine had been five to six-times the IRPs at the privately owned pharmacies and four to seven-times at the *Rajya Osu Sala* over the 6-years period. MPR of below 2.5 is considered efficient pricing in the private sector.[11] Therefore, the innovator product pricing had been higher than the efficient pricing both in the privately owned pharmacies and in the *Rajya Osu Sala* throughout the study period.

However, the median MPRs for innovator products were lower in Sri Lanka compared with Malaysia (median MPR of 16.35 for innovator products in private sector retail pharmacies).[12] But, the innovator product pricing in Sri Lanka is not as efficient as that reported from six survey areas in India, where the median MPRs of INOs varied from 1.74 to 4.38, with only the state of Maharashtra (four regions) reporting a MPR above 3 and the state of Karnataka and Maharashtra (12 districts) reporting MPRs between 2.5 and 3.[13]

In contrast, the generic pricing in Sri Lanka had been very efficient. In all 6 years, both in privately owned pharmacies and *Rajya Osu Sala*, the prices of most sold generic and cheapest generics had been ≤1 the IRP. In comparison, Malaysia reported median MPRs for MSGs and CGs as 6.89 and 6.57, respectively, and in the six regions of India reporting median MPRs of 1.3-1.69 for MSGs and 1.3-1.84 for CGs.[12,13]

Therefore, the prices of generics in Sri Lanka have consistently been efficient. Our generic pricing would indeed facilitate the affordability and equity of medicine to the majority of the patients. The reason for the lower pricing for generic medicine is not very clear; however, this may be due to State Pharmaceuticals Corporation’s dominance in the market and their procurement system.

#### Change in prices

In terms of change in medicine prices over the 6 years, there is no clear pattern in the change of prices except for the prices of most sold generics in the *Rajya Osu Sala*, which shows an increase. The MPRs of innovator products and cheapest generics have increased in 2006 and decreased in 2009 compared with the MPRs of 2003 in the privately owned pharmacies. In the *Rajya Osu Sala*, the MPRs of INOs and CGs have increased in 2006 and 2009 compared with the MPRs of 2003; however, there was a drop in the MPRs from 2006 to 2009.

#### Variation of prices among pharmacies

Because the pricing formula is not strictly implemented, currently, the prices of individual medicines could be decided by the importers, manufacturers and individual pharmacy owners. According to our study, the median MPR of medicines did not vary considerably among the pharmacies within the geographic area surveyed. In an interview with the State Pharmaceuticals Corporation, the operator of the *Rajya Osu Sala*, it was found that the corporation fixes its prices following the pricing formula, despite its nonimplementation. Even though healthcare markets do no operate following competitive market rules, it appears that the small privately owned pharmacy owners tend to fix a competitive price compared with that of the *Rajya Osu Sala* outlets. This could be the most likely reason for the low variation in prices of medicines among the pharmacies surveyed.

#### Brand premium

It was also observed that although the MPRs for innovator and generic products have changed during the three study periods, for certain products, the ratio between the MPRs of the innovator products and generics (brand premium) were the same for the years 2003 and 2009. This might be because a change in the prices of innovators and generics followed a similar pattern of multiplication.

#### Availability

In terms of availability, 50–80% is considered as “fairly high” and above 80% is considered as “high” availability.[11] Therefore, innovator products had a fairly high availability and the most sold generics had a fairly high to a high availability while the cheapest generics were constantly high in availability throughout the study period.

The high availability for cheapest generic products in all 6 years may reflect the purchasing power of the majority of the patients. Innovator products of certain medicine were not available all through the study period. These products might have not been imported into the country due to lack of demand.

#### Affordability

Our affordability data indicate that innovator brands always cost more than a days wage of the lowest paid government worker (except for amoxicillin in 2006 and 2009). But, affordability of generic products had always been less than one day’s wage, causing fewer issues to the patients in Sri Lanka. This reflects well for Sri Lanka even without any price control. There seems to be no difference in affordability whether the place of purchase is privately owned or semigovernment pharmacy, *Rajya Osu Sala*.

## Conclusion

In Sri Lanka, generic medicines have effective pricing and are available and affordable. In contrast, innovators are higher than the effective pricing and are not affordable. No drastic changes in prices of medicine in the private sector were observed over the 6 years despite not having a price control. No marked differences in pricing were observed in *Rajya Osu Sala* and privately owned pharmacies. The high availability of generic products at all times ensures affordability and equity for patients.

## References

[1] (2003). Central Bank of Sri Lanka. Annual Report 2003.

[2] Balasubramaniam K, Jayasekara J (2009). Affordability and Medicine’s Prices in Sri Lanka. Paper presented at: Technical Sessions of the Pharmaceutical Society of Sri Lanka 2009.

[3] (2003). World Health Organization, Health Action International. Medicines prices: A new approach to measurement.

[4] Angell M (2004). The Truth About the Drug Companies how they deceive us and what to do about it.

[5] Aaserud M, Austvoll-Dahlgren A, Kösters JP, Oxman AD, Ramsay C, Heidrun S Pharmaceutical policies: Effects of reference pricing, other pricing, and purchasing policies. 2006 Apr 19. In: Cochrane Database Systemic Reviews [Internet].

[6] Weerasuriya K, Amarasekeara N (1999). List of Essential Drugs: Second Revision. Colombo: Ministry of Health Sri Lanka, Sponsored by the State Pharmaceuticals Manufacturing Corporation of Sri Lanka.

[7] The international price indicator guide [Internet]. Massachusetts: Management Sciences for Health.

[8] Central Bank of Sri Lanka [Internet].

[9] Central Bank of Sri Lanka [Internet].

[10] Central Bank of Sri Lanka [Internet].

[11] Gelders S, Ewen M, Noguchi N, Laing R (2006). Price, availability and affordability: An international comparison of chronic disease medicines.

[12] Babar ZU, Ibrahim MI, Singh H, Bukahri NI, Creese A (2003). Evaluating drug prices, availability, affordability, and price components: Implications for access to drugs in malaysia. PLoS Med.

[13] Kotwani A, Ewen M, Dey D, Iyer S, Lakshmi PK, Patel A (2007). Prices and availability of common medicines at six sites in India using a standard methodology. Indian J Med Res.

